# Post-Mastectomy Pain Syndrome: Defining Perioperative Etiologies to Guide New Methods of Prevention for Plastic Surgeons

**DOI:** 10.29252/wjps.9.3.247

**Published:** 2020-09

**Authors:** Ava G Chappell, Jennifer Bai, Selcen Yuksel, Marco F Ellis

**Affiliations:** Division of Plastic and Reconstructive Surgery, Northwestern Medicine, Feinberg School of Medicine, Chicago, IL, USA

**Keywords:** Mastecomy, Breast neoplasms, Chronic pain, Post operative, Plastic surgery, Reconstructive surgical procedures

## Abstract

From discussing the etiologies of post-mastectomy pain syndrome and potential methods of prevention, the next step is to create specific methods of prevention and to identify ways to measure their effects. With the increase in breast cancer related surgeries and increased survival after breast cancer patients, efforts must be made to prevent chronic pain and improve quality of life for these patients after surgery. The plastic surgeon, skilled in breast reconstruction and peripheral nerve reconstruction, may play a significant role in eliminating chronic pain after breast cancer related surgery.

## INTRODUCTION

More than 1.7 million new cases of breast cancer are diagnosed every year in women worldwide.^1^ Following mastectomy and breast cancer related procedures, an estimated 25-60% of patients suffer from chronic pain are often referred as post-mastectomy pain syndrome (PMPS) cases.^2^^-^^4^ Treatments include oral analgesics, topical ointments, antidepressants, scar revision, and fat grafting. However, efficacy of these treatments varies, and many continue to suffer from chronic pain, so prevention is optimal. To date, no standardized methods to prevent PMPS exist. The current lack of understanding of PMPS prohibits successful treatment and prevention.^2^^-^^4^ In this study, we present the main etiologies of PMPS and discuss potential methods of prevention to guide future clinical studies. 

**Defining PMPS or chronic pain after breast cancer related surgery**

Different definitions and terms related to PMPS exist in the literature (Table 1). Chronic pain after mastectomy was originally reported in 1978 entitled “Intercostobrachial nerve entrapment syndrome”.^5^ In 1987, the term was coined as “Post-mastectomy pain syndrome” to represent pain and sensory changes specifically following mastectomy.^6^ In 2003, post-mastectomy neuropathic pain was further classified into four categories of phantom breast pain, intercostobrachial neuralgia from damage to intercostobrachial nerve (ICBN), neuroma pain provoked by percussion, and other nerve injury pain from damage to surrounding nerves.^7^

**Table 1:** Definitions in the literature used to describe chronic pain after breast cancer related surgery

**Author**	**Year**	**Term**	**Definition**	**Citation**
Wood	1978	Intercostobrachial nerve entrapment syndrome	Complication of breast surgery… syndrome reproduced by putting pressure on a point just below the second rib, close to the anterior axillary line	Wood KM. Intercostobrachial nerve entrapment syndrome. *South Med J.* 1978; 71(6):662-663.
Granek, et al.	1984	Post-mastectomy pain syndrome (PMPS)	Distinct syndrome of pain and sensory abnormalities following mastectomy	Granek I, Ashikari R, Foley K. The post-mastectomy pain syndrome: Clinical and anatomical correlates. *Proc Am Soc Clin Oncol.* 1984; 3(1):122.
Jung, et al.	2003	Post-mastectomy neuropathic pain	Classified into four categories: 1) Phantom breast pain; 2) Intercostobrachial neuralgia due to damage to the Intercostobrachial Nerve (ICBN) presenting as pain and sensory changes localized to the axilla, medial upper arm, and/or anterior chest wall; 3) Neuroma pain in the region of scar on the breast, chest, and/or arm, provoked by percussion; 4) Other nerve injury pain resulting from damage to the medial or lateral pectoral, long thoracic, or thoracodorsal nerves	Jung BF, Ahrendt GM, Oaklander AL, Dworkin RH. Neuropathic pain following breast cancer surgery: Proposed classification and research update. *Pain*. 2003; 104(1-2):1-13.
Vilholm, et al.	2008	Post-mastectomy chronic pain (PMCP)	Pain localized in the area of the surgery or in the ipsilateral arm, present at least 4 days per week and with an average intensity of at least 3 on a numeric rating scale from 0-10	Vilholm OJ, Cold S, Rasmussen L, Sindrup SH. The postmastectomy pain syndrome: An epidemiological study on the prevalence of chronic pain after surgery for breast cancer. *Br J Cancer*. 2008; 99(4):604-10.
Andersen, et al.	2013	Chronic pain after breast cancer treatment	Neuropathic pain condition localized in and around the area of surgery and lasting more than 3 months after surgery	Andersen KH, Kehlet H. Persistent pain after breast cancer treatment: A critical review of risk factors and strategies for prevention. *J Pain*. 2011; 12:725-46.
International Association for Study of Pain (IASP)	1986	Post mastectomy chronic pain (PMCP)	Chronic pain in the anterior aspect of the thorax, axilla, and/or upper half of the arm beginning after mastectomy or quadrantectomy and persisting for more than three months after the surgery	International Association for the Study of Pain (IASP) Classification of chronic pain. Description of chronic pain syndromes and definitions of pain terms. The International Association for the Study of Pain, Subcommittee on Taxonomy. *Pain*. 1986; 3:51-226.
Belfer, et al.	2013	Persistent post- mastectomy pain (PPMP)	Persistent levels of breast pain in first 6 months following surgery	Belfer I, Schreiber KL, Shaffer JR, Shnol H, Blaney K, Morando A, Englert D, Greco C, Brufsky A, Ahrendt G, Kehlet H, Edwards RR, Bovbjerg DH. Persistent post-mastectomy pain in breast cancer survivors: Analysis of clinical, demographic and psychosocial factors. *J Pain*. 2013; 14:1185-95.
Waltho, et al.	2016	Post-mastectomy pain syndrome (PMPS)	Pain that occurs after any breast surgery; at least moderate severity; neuropathic qualities; located in ipsilateral breast/chest wall, axilla, arm; lasts at least 6 months’ occurs at least 50% of the time; may be exacerbated by movement of shoulder girdle	Waltho D, Rockwell G. Post-breast surgery pain syndrome: Establishing a consensus for the definition of post-mastectomy pain syndrome to provide a standardized clinical and research approach – A review of the literature and discussion. *Can J Surg.* 2016; 59(5):342-50.

More recently, “Post-breast surgery pain syndrome (PBSPS)” was proposed as a more accurate name for PMPS and defined as PBSPS: Pain that occurs after any breast surgery; to be at least with moderate severity; to possess neuropathic qualities; to be located in the ipsilateral breast/chest wall, axilla, and/or arm; to last at least 6 months; to occur at least 50% of the time; and to be exacerbated by movements of the shoulder girdle.^8^ However, the current definition for PMPS used by the International Association for Study of Pain (IASP) is “chronic pain in the anterior aspect of the thorax, axilla, and/or upper half of the arm beginning after mastectomy or quadrantectomy and persisting for more than three months after the surgery”,^9^^-^^11^ which is the definition of PMPS that we will use in this review. 

**Etiologies of PMPS**

**Fig. 1:**
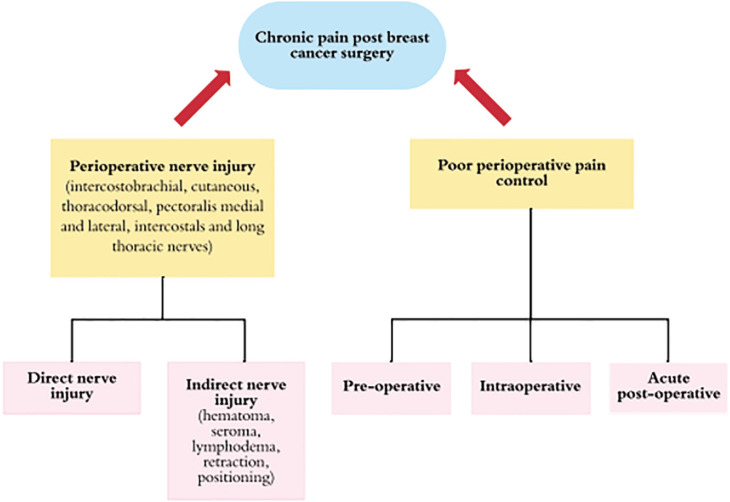
Diagram demonstrating perioperative etiologies of chronic pain after breast cancer related surgery, also known in the literature as post mastectomy pain syndrome

The current established perioperative risk factors for PMPS are injuries to nerves in the axilla/chest wall, axillary lymph node dissection (ALND) and inadequate control of perioperative pain (Figure 1). The main nerves at risk during mastectomy are: ICBN, medial and lateral pectoral, thoracodorsal, long thoracic and intercostal nerves.^12^^,^^13^ Nerve injury can be direct via transection (neurotmesis), or indirect from traction, compression or scar adhesion (neuropraxia, axonotmesis). Direct nerve injury most often occurs with ALND and radical mastectomy. ALND is a major risk factor for PMPS, as the ICBN is often resected or injured.^8^^,^^12^^-^^20^

Indirect nerve injury can occur intra-operatively or postoperatively. Intraoperatively, retraction and poor arm positioning can stress and compress peripheral nerves.^21^ Post-operatively, stretch and compression injuries can occur from hematoma, seroma and scarring. Case reports have shown that aggressive aspiration of seromas and hematomas in post mastectomy patients can successfully treat PMPS.^22^ Other studies have examined the use of fat grafting to soften scars, decrease nerve entrapment within adhesions, and provide analgesia by inhibiting the local inflammatory response.^23^ Of note, radiation therapy has been associated with PMPS. While the cause is not well understood, radiation fibrosis and chronic inflammation can cause nerve entrapment.^10^

Poorly controlled perioperative pain also increases risk of developing PMPS. The anesthesia literature supports the use of local and regional blocks to prevent PMPS by providing better control of intraoperative and acute postoperative pain.^24^ Women who experienced moderate to severe acute postoperative pain and required medication were shown to be a good predictor of PMPS.^24^^,^^25^ Others have reported the association of greater severity of postoperative pain and increased consumption of analgesics with PMPS (Figure 1).^10^^,^^26^^-^^29^

## DISCUSSION

The etiologies mentioned above now led us to discuss potential methods of prevention of PMPS, which can be implemented by plastic surgeons. Operative techniques and standardized guidelines to protect vulnerable nerves during mastectomy and ALND are needed to decrease direct nerve injury with attention to protecting the ICBN. While this may not always be possible due to location and extent of tumor burden, increased awareness of the location of these nerves may lead to decreased incidence of direct nerve injury and PMPS. Furthermore, when nerves are clearly transected during ALND or mastectomy, measures to prevent neuroma formation in the proximal nerve segment may be employed. Specific methods to treat neuroma pain described in the plastic surgery literature include targeted muscle reinnervation (TMR), regenerative peripheral nerve interfaces (RPNIs) and nerve reconstruction with allograft.^30^^-^^32^

TMR, or rather here, targeted sensory nerve re-innervation by coapting the transected nerve ending to a local motor nerve or sensory nerve branch may prevent chronic neuroma pain after mastectomy and/or ALND. Another method to investigate would be securing the proximal nerve ending to local muscle, known as a regenerative peripheral nerve interface (RPNI). Finally, nerve allograft could be used as interposition grafts to proximal and distal segments in close proximity. Further prospective clinical studies will examine the outcomes of these techniques in decreasing chronic pain after breast cancer surgery.^23^

To minimize indirect nerve injury, close attention to postoperative seromas and hematomas is warranted. Limiting inflammation and scarring in certain locations with silicone, scar massage, and/or fat grafting may decrease incidence of PMPS. Careful positioning and retraction should also be practiced, to reduce risk of stretch and compression injury to nerves. To optimize perioperative pain management, breast surgeons and plastic surgeons must collaborate with anesthesiologists to help determine methods to decrease the incidence of PMPS. Intraoperative preventative methods include the use of local anesthesia and regional blocks including thoracic epidural or spinal anesthesia, thoracic paravertebral block, and ultrasound-guided interfascial plane blocks such as pectoral nerve blocks and serratus plane block.^23^

Standardized use of local and regional blocks may decrease the incidence of PMPS. For postoperative pain management, combining regional blocks with prescription analgesia regimens may reduce severe postoperative pain and decrease risk of developing PMPS. It is imperative that the breast oncology surgeon, the plastic surgeon and the anesthesiologist, coordinate an analgesic plan is the most therapeutic for the breast cancer patient in the post-operative acute and long-term periods. 

## CONCLUSION

From discussing the etiologies of PMPS and potential methods of prevention, the next step is to create specific methods of prevention and to identify ways to measure their effects. Anatomical studies of the breast can demonstrate specific nerves and their locations at risk during breast cancer surgery to provide a map for breast and plastic surgeons to decrease risk of direct and indirect nerve damage intraoperatively. There may be a role for targeted muscle reinnervation (TMR) and/or regenerative peripheral nerve interfaces (RPNIs) to prevent neuroma formation after nerve transection during ALND and/or mastectomy. Clinical prospective studies are needed to examine the pain outcomes after nerve transfer or nerve reconstruction that occurs during breast cancer related procedures. 

Innovations in preventing post-mastectomy pain also apply to breast reconstruction. With increasing use of prepectoral tissue expanders, further studies examining the differences in chronic postoperative pain outcomes in prepectoral versus subpectoral reconstruction patients who both undergo ALND should be examined. The role of acellular dermal matrices in decreasing risk of chronic pain after mastectomy also remains to be explored. With the increase in breast cancer related surgeries and increased survival after breast cancer patients, efforts must be made to prevent chronic pain and improve quality of life for these patients after surgery. The plastic surgeon, skilled in breast reconstruction and peripheral nerve reconstruction, may play a significant role in eliminating chronic pain after breast cancer related surgery. 
